# Nosocomial infection in a newborn intensive care unit (NICU), South Korea

**DOI:** 10.1186/1471-2334-6-103

**Published:** 2006-06-23

**Authors:** Ihn Sook Jeong, Jae Sim Jeong, Eun Ok Choi

**Affiliations:** 1 College of Nursing, Pusan National University, Busan, South Korea; 2 Department of Clinical Nursing, College of Medicine, University of Ulsan, Seoul, South Korea; 3 Department of Nursing, College of Medicine, Inje University, Busan, South Korea

## Abstract

**Background:**

This study aimed to determine the occurrence of nosocomial infections (NIs), including infection rates, main infection sites, and common microorganisms. Patients included in the study were taken from a newborn intensive care unit (NICU), in a hospital in South Korea.

**Methods:**

A retrospective cohort study was performed by reviewing chart. The subjects were 489 neonates who were admitted to the NICU, survived longer than 72 hours, and not transferred to another unit, between Jan. 1. 1995 to Sep. 30, 1999. NIs were identified according to the NNIS definition. Data were analyzed with descriptive statistics.

**Results:**

Cumulative incidence rate for NIs was 30.3 neonates out of 100 admissions, with a total of 44.6 infections. The incidence density was average 10.2 neonates and 15.1 infections per 1000 patient days. The most common infections were pneumonia (28%), bloodstream infection (26%), and conjunctivitis (22%). Major pathogens were Gram-positives such as *Staphylococcus aureus *and coagulase-negative staphylococci. The factors associated with NI was less than 1500 g of birth weight, less than 32 weeks of gestational age, and less than 8 of apgar score. There's no statistical difference in discharge status between two groups, but hospital stay was longer in subjects with nosocomial infection than those without infection.

**Conclusion:**

Although the distribution of pathogens was similar to previous reports, a high rate of nosocomial infection and in particular conjunctivitis was observed in this study that merits further evaluation.

## Background

Even though recent marked advances in intensive care skill and facilities have improved the survival rate of high risk neonates, they have also increase the frequency of invasive procedures. Because most of neonates in newborn intensive care units (NICUs) are premature and highly susceptible to infection due to immaturity of immune function and impaired defense mechanism, this frequent invasive procedures can easily cause the infections. Therefore, nosocomial (hospital-acquired) infections are an important and critical issue related to high morbidity and mortality in high risk neonates [1,2].

For the above reasons, many studies worldwide have been done to identify the epidemiology of the nosocomial infections among neonates in NICUs [3-18]. However studies on the epidemiology of the nosocomial infections among neonates in South Korea were very few, studies on neonatal infections have been focused on the sepsis and even hospital acquired infection and community acquired infections were not differentiated in those studies [19,20].

Therefore, this study was conducted in order to investigate the epidemiological characteristics of nosocomial infections in the aspects of infection rate, common infection sites and pathogens in an NICU of South Korea.

## Methods

### Study subjects

This study was performed in a 20- bed NICU with an average of 160 annual admissions. This NICU is an urban, university affiliated, multidisciplinary, referral center of 1100-bed teaching hospital in Seoul, South Korea. The NICU consists of 1 room with limited available space, average 5 nurses, and backed up by one or two neonatologists for 24-hours a day.

### Data collection

The information on nosocomial infection was collected retrospectively by the researcher and an infection control practitioner of the study hospital. The researcher designed a specific form to gather the information on nosocomial infections; number of infection, infection site, occurrence date, pathogens. All charts and related records including laboratory data, medical and nursing records were examined and recorded on the specific form by the researcher. Hospital acquired (nosocomial) infections were defined in accordance with the National Nosocomial Infection Surveillance (NNIS) definition of Centers for Disease Control and Prevention [21]. This study was not required specific approval by the Ethics Committee since data used for the study had already been collected for clinical purposes.

### Statistical analysis

Statistical analyses were performed using descriptive statistics such as frequency and percentage, or mean and standard deviation. The infection rates were calculated with the number of infection per 100 admitted neonates (cumulative incidence rate) and number of infection per 1000 patient days (incidence density).

## Results

### Study population and survey rate

Total 696 neonates were admitted after being born in this hospital from Jan. 1, 1995 to Sep. 30, 1999. Patients readmitted during the study period were included only at the time of their first admission. Among them, 35 neonates were died, discharged or transferred within the 72 hours and 172 neonates couldn't be accessed because of unavailable chart were excluded and finally 489 neonates who survived longer than 72 hours after admission were selected as study subjects.

### Characteristics of study subjects

Male was 51%, mean birth weight was 1983 g, mean gestational age was 33.6 weeks, average Apgar score at 1 minute and 5 minute after birth was 5.8 and 7.2 respectively, and over 62% of neonates were born by Caesarean section. Most of subjects belonged to pediatrics department and the major diagnosis was prematurity (61.6%). The median hospital stay was 22.4 days (Table 2).

**Table 1:** Study population and survey rate

	No. of admission (1)	Died, discharged or transferred within the 72 hours(2)	Chart not available (3)	final study population (4)	survey rate (4)/(1)x100 (%)
95. 1. 1 ~ 12. 31	160	3	89	68	42.5
96. 1. 1 ~ 12. 31	150	6	36	108	72.0
97. 1. 1 ~ 12. 31	142	12	18	112	78.9
98. 1. 1 ~ 12. 31	119	5	15	99	83.2
99. 1. 1 ~ 9. 30	125	9	14	102	81.6

Total	696	35	172	489	70.3

**Table 2:** General characteristics of study subjects

	Characteristics	N	%	Mean ± SD
Gender				
	Male	250	51.1	
	Female	239	48.9	
Birth weight (g)				
	>2500	109	22.3	
	1501–2500	245	50.1	
	1001–1500	100	20.4	
	≤1000	35	7.2	1983 ± 773
Gestational age (weeks)				
	≥37	92	18.8	
	32–36	255	52.1	
	28–31	113	23.1	
	≤27	29	5.9	33.6 ± 3.6
Apgar score at 1 minute				
	≥ 8	138	28.2	
	4–7	259	53.0	
	< 4	92	18.8	5.8 ± 2.4
Apgar score at 5 minute				
	≥ 8	239	48.9	
	4–7	228	46.6	
	< 4	22	4.5	7.2 ± 1.7
Delivery type				
	Vaginal	182	37.2	
	Caesarean Section	307	62.8	
Medical department				
	Pediatrics	448	91.6	
	Neurosurgery	7	1.4	
	General surgery	34	7.0	

Diagnosis				
	Hyaline membrane disease	63	12.9	
	Prematurity	301	61.6	
	Congenital anomaly	112	22.9	
	Others*	13	2.6	
Discharge status				
	Live to home	409	83.6	
	Live to other hospitals	36	7.4	
	Against medical advice(AMA)	24	4.9	
	Death	21	4.3	
Hospital stay(day)				29.6 ± 23.9(median : 22.4)

* others : carcinogenesis 6, meconium aspiration syndrome 3, chromosomal abnormality 3, facial palsy 1

### Incidence of nosocomial infection

Two hundred and eighteen hospital-acquired infections from one hundred forty eight neonates were identified, and 64.2% among 148 infected patients had a singleinfection. Cumulative incidence rates were 30.3 neonates and 44.6 infections per 100 admitted neonates and the incidence densities were 10.2 neonates per 1000 patient days (Table 3).

**Table 3:** Incidence of nosocomial infection

Total number of newborn included	489
Total patient days	14,451 days
	
Neonates infected	148
Nosocomial infections (NI)	218
Number of NI per neonates infected	
1	95(64.2%)
2	40(27.0%)
≥ 3	13(8.8%)
	
Infected patients/100 neonates	30.3
Infections/100 neonates	44.6
	
Infected patients/1000 patient days	10.2
Infections/1000 patient days	15.1

### Median time from admission to development of nosocomial infection

Median time to nosocomial infection from NICU admission was 16 days, with range of 3–118 days. It took 15 days until occurring pneumonia, 17 days for bloodstream infection, and 16 days for conjunctivitis (Table 4).

**Table 4:** The time to infection from ICU admission

	From admission to onset of infection (days)		
	
Major site	Minimum	Median	Maximum
Pneumonia	3	15	41
Bloodstream infection	4	17	96
Conjunctivitis	3	16	118
Urinary tract infection	6	18	70
Gastrointestinal infection	3	11.5	42
Cardiovascular infection (Venous infection)	5	10	47
Surgical site infection	3	15	36
Other sites	8	24.5	109

Total	3	16	118

### Sites of nosocomial infection

**Figure 1:**
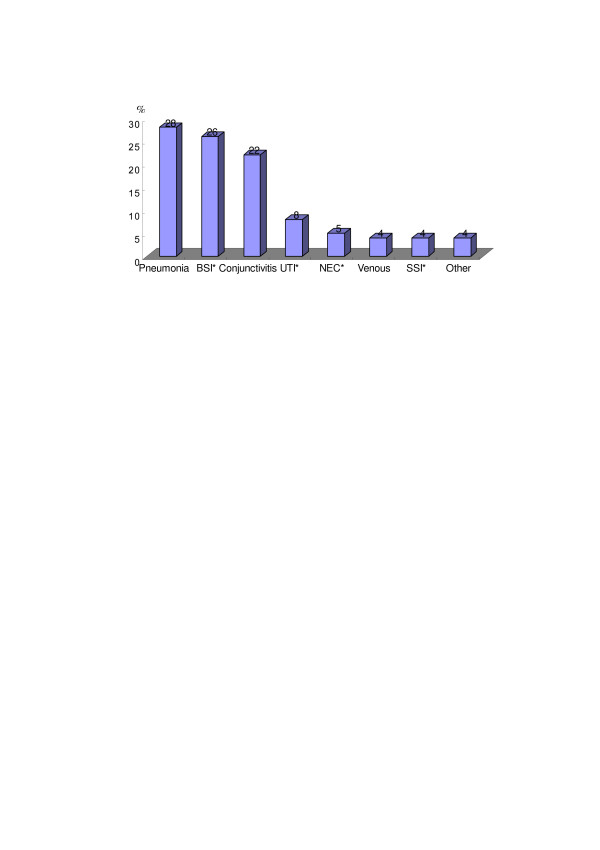
**Distribution of infection site. **BSI : bloodstream infection, UTI : Urinary tract infection, NEC : necrotizing enterocolitis, SSI : surgical site infection

The most common infection site was pneumonia (60 cases, 28%) followed by bloodstream infection (56 cases, 26%) and conjunctivitis (49 cases, 22%) (Figure 1).

### Distribution of pathogens by major infection sites

The most frequently isolated organism was *S. aureus*, followed by coagulase-negative *staphylococcus*. Overall Gram-positives (60.2%) were more common than Gram-negatives (31.8%). While *S. aureus *was the most common cause of pneumonia and bloodstream infection, coagulase- negative *staphylococcus *was the most common pathogen of conjunctivitis.

### Characteristics of study subjects with or without nosocomial infection

Factors associated with the development of nosocomial infection were birth weight, gestational age and Apgar score at 1 min or 5 min (Table 6). Less than 1500 g of birth weight, less than 32 weeks of gestational age, and less than 8 of Apgar score increased the risk of nosocomial infection.

**Table 5:** Distribution of pathogens by major infection sites

Type	Name of pathogens	PNEU^1)^	BSI^2)^	CON^3)^	UTI^4)^	Total (%)
G(+)	*S. aureus*	21	12	15	2	50 (28.4)
	CoNS^5)^	8	9	20	0	37 (21.0)
	*Enterococcus *spp	1	1	1	0	3 (1.7)
	S. viridans group	13	0	2	0	15 (8.5)
	*S. pneumoniase*	1	0	0	0	1 (0.6)
	Subtotal	44	22	38	2	**106 (60.2)**
						
G(-)	*Enterobacter *spp.	5	3	0	5	13 (7.4)
	*E. coli*	2	1	2	3	8 (4.5)
	*P. aeruginosa*	8	0	4	0	12 (6.8)
	*K. pneumoniae*	7	0	2	5	14 (8.0)
	*Acinetobacter *spp.	5	1	2	1	9 (5.1)
	Subtotal	27	5	10	14	**56 (31.8)**
						
Fungi	*C. albicans*	1	6	1	2	10 (5.7)
	*C. parapsilosis*	0	0	0	1	1 (0.6)
	Subtotal	1	6	1	3	**11 (6.3)**
						
Others^6)^		1	1	1	0	3 (1.7)
Total		73	34	50	19	176 (100.0)

1) PNEU : pneumonia, 2) BSI : bloodstream infection, 3) CON : conjunctivitis, 4) UTI : Urinary tract infection, 5) CoNS : coagulase-negative *staphylococcus *6) Others : Chlamydia for Pneumonia, *P. cepacia *for BSI, Bacillus spp. for conjunctivitis, and enterovirs for others

**Table 6:** Characteristics of study subjects with or without nosocomial infection

	Nosocomial infection			
			
Characteristics	Yes (n = 148)	No (n = 341)	OR(95% CI)	p
Gender				
Male	81(54.7)	169(49.6)	1	
Female	67(45.3)	172(50.4)	0.81 (0.55–1.20)	0.293
Birth Weight (g)				
>2500	24(16.2)	85(24.9)	1	
1501–2500	63(42.6)	182(53.4)	1.23(0.72–2.10)	0.456
1001–1500	40(27.0)	60(17.6)	2.36(1.29–4.32)	0.005
≤1000	21(14.2)	14(4.1)	5.31(2.35–11.99)	<0.0001
Gestational age (weeks)				
≥37	19(12.8)	73(21.4)	1	
32–36	61(41.2)	194(56.9)	1.21(0.68–2.16)	0.523
28–31	50(33.8)	63(18.5)	3.05(1.63–5.71)	<0.0001
≤27	18(12.2)	11(3.2)	6.29(2.55–15.53)	<0.0001
Apgar score at 1 min				
≥ 8	25(16.9)	113(33.1)	1	
4–7	82(55.4)	177(51.9)	2.09(1.26–3.47)	0.004
< 4	41(27.7)	51(15.0)	3.63(2.00–6.60)	<0.0001
Apgar score at 5 min				
≥ 8	48(32.4)	191(56.0)	1	
4–7	87(58.8)	141(41.3)	2.46(1.62–3.72)	<0.0001
< 4	13(8.8)	9(2.6)	5.75(2.32–14.24)	<0.0001
Delivery type				
V/D^1)^	57(38.5)	125(36.7)	1	
C/S^1)^	91(61.5)	216(63.3)	0.92 (0.62–1.38)	0.696
Discharge status				
Live	129(93.9)	315(92.4)	1	
AMA^2)^ or death	19(6.1)	26(7.6)	1.78(0.95–3.34)	0.067
Hospital stay(days)				
Mean ± SD	44.4 ± 27.6	23.1 ± 18.9		<0.0001

^1)^ V/D : vaginal delivery, C/S : cesarean section.

^2)^ AMA : against medical advice

However, the outcome of care was compared between study subjects with or without nosocomial infection in the aspects of discharge status and hospital stay. There's no statistical difference in discharge status between two groups, but hospital stay was longer in subjects with nosocomial infection than those without infection.

## Discussion

This study was designed to investigate the epidemiological characteristics of nosocomial infections (NIs) regarding the infection rate, common infection sites and pathogens in an NICU of South Korea.

According to previous reports, the incidence of NIs has been reported from 6.2 to 50.7 infections per 100 admissions or discharges, and from 4.8 to 62 infections per 1000 patient days [3,14,15,17,21,23,24]. In this study, the incidence of NIs was 44.6 infections per 100 admissions, and 15.1 infections per 1000 patient days. Even though this result shows the incidence rate is very high compared to previous studies, caution should be taken when comparing infection rates among studies due to the differences in the place where the studies were performed, and the methodology to be used. We should consider a few factors regarding the incidence rate of this study.

First, there's a difference in the surveillance method for NIs. Many studies including this study [3,10,14,23,24], diagnosed NIs based on the clinical and culture-proven evidence according to the CDC definition of NIs; however, Efird et al. (2005) used culture-proven evidence only [15].

Second, there's a difference in the length of stay for patients in different institutions. It may be misleading to compare such rates without being aware of the difference in the average length of stay at different institutions.^10) ^Interestingly, the incidence rate is very high but the incidence density is not so high, which may be related to the longer length of hospital stay of the subjects in this study. Length of stay can be related to the subjects' unique conditions, such as birth weight or gestational age. The lower birth weight or gestational age they have, the more infections they have [4,5,10,11]. The mean hospital stay of this study's infants was about 30 days, compared to more than 12.4 days in Drews et al. (1995) and van der Zwet et al. (2005) [3,17].

Third, there were a few small sized epidemics of conjunctivitis during the study period. Most previous studies reported that the main sites of infection in neonates were bloodstream infections or pneumonia, even though the order was different study by study. The largest infection was pneumonia in this study, which is similar to the previous studies. In general, the incidence of conjunctivitis has been reported to be about 5% [14,16], but twice higher (49 cases, 10.0%) in this study. However, in the recent study of Mireya et al. (2006) [18], the conjunctivitis was 20% as the second common NICU infection. The high percentage of coagulase-negative *staphylococci *and *S. aureus *isolated suggests that this may result from repeated handling of the patients by health care professionals and their families with poor handwashing [25], or it may be an artifact due to the ambiguity of diagnosis and intense surveillance. Because a few Epidemic Kerato-conjunctivitis (EKC) epidemics have been documented in study NICUs [26], we diagnosed most patients who have pathogens cultured from purulent exudates from the conjunctiva or redness with purulent exudates as conjunctivitis based to the NNIS definition [21]. But a few cases among them might be just eye infections, not conjunctivitis.

Fourth, though this can be applied not only to this study, but also others use the NNIS definition to make diagnoses of the occurrence of NIs. Because of a lack of standardized definitions of NIs in NICUs, the NNIS definition of CDC is regarded as the best available for surveillance. However, as pointed out by van der Zwet et al (2005), CDC definitions of BSI for children aged < 1 year were somewhat unsuitable for preterm neonates. Under the NNIS definition [17], we can diagnose BSI by laboratory confirmation or through symptomatology such as irritability apathy, bradycardia, tachycardia, hyperthermia with a temperature of more than 38.0°C, or hypothermia with a temperature of less than 37.0°C. However, it is difficult to get the appropriate volume of blood, and some physicians didn't consult blood culture. Therefore, the incidence rate of laboratory confirmed BSI could be reduced because of a lack of evidence of blood culture or the inaccuracy of a relatively small volume of blood for a culture [24].

The common infection sites were similar to previous studies except for conjunctivitis. Bloodstream infection and pneumonia are the most common NIs reported in the literature [3,5,7,10,14,15], with the following rates : 16% to 78% for bloodstream infections, and 10% to 40% for pneumonia. The common type of NI was strongly related to the use of invasive devices [4,11].

Over 60% of all isolates were Gram-positive cocci (GPC) and about 32% were Gram-negative rods (GNR). Even though the proportions are somewhat different, most of previous studies have shown that Gram-positive bacteria are more than Gram-negative one [3,5,7,18]. But according to the study by Mambiar et al (2002) conducted from 1996 to 2001, the proportion of the GNR was 42.7% and the GPS was 33.5% [12], and it showed the predominance of GNR. This change seems to be related to the increasing antimicrobial resistance among Gram-negative bacteria and may thus be contributing to the reemergence of GNR as a dominant pathogen. Considering the change of distribution of pathogens by time, further regular studies should be performed to confirm this result and to help choose antimicrobials for the empiric treatment of hospital acquired infections in neonates. The most common pathogen of pneumonia was *S. aureus*, which is the same result of the large scale study of Richards et al (1999) [7]. For bloodstream infection, while the dominant pathogen was *S. aureus *in our study, coagulase-negative staphylococci was major inRichards et al (1999) and Mireya et al (2006) [7,18].

Premature and low birth weight infants have shown significantly higher risk for developing infections, and this result is in agreement with the literature available on NICU [8,11,13,18]. The lower gestational age and birth weight they are, the higher risk of NIs are.

The advantage of this study is that it was the first to identify the epidemiological characteristics of nosocomial infections in an NICU in South Korea, but there are a few limitations in the areas of study design and study subjects. The problems were as follows:

First, because this study used a retrospective design, the incidences of some infections such as oral cavity infections and venous infections could be underestimated. Oral cavity infections couldn't be diagnosed when there were no records or documents about the occurrences in the charts. According to the clinical experience of researchers, oral cavity infections were common in this NICU. Nonetheless, there were some cases in which the signs and symptoms or characteristics of oral cavity were not mentioned in their charts and there were others that oral thrush had been treated with Gentian violet, but no reports on the signs and symptoms or laboratory results. In these cases we couldn't make a diagnosis of oral cavity infections. This is true of venous infections.

Second, the study subjects were selected from only one NICU in Seoul, even though this NICU has a long history and is representative of NICUs in South Korea. Therefore, generalizability of the findings can be limited. To increase generalizability of the findings, more NICUs from a broad geographic area must be studied.

Third, the rate of missing charts was very high. All charts for the study subjects were stored in and managed by the Department of Medical Record (DMR). The 72.7% (152/172) of unavailable chart was recorded in 1995 and 1996. During the data collecting period from Sep. to Dec. 1999, the office of DMR had been moved to the temporary building because of renovation of the original office. So the older charts had not been arranged well. We repeatedly requested to track and find the unavailable charts to the officer of the Department of Medical Record, but it was difficult to track some missing charts of the study subjects. We tried to track and review all charts of the study subjects, but failed, because of time limitation. We thought that it would be enough to represent the characteristics nosocomial infections because the records since 1997 were almost complete.

## Conclusion

The distribution of pathogens and risk factors of nosocomial infection in NICU were similar to previous studies. The rate of conjunctivitis was very high compared to the previous studies, but one of the recent studies showed the same result. Further studies are needed to investigate the trend of the incidence of conjunctivitis and related factors.

## Competing interests

The author(s) declare that they have no competing interests.

## Authors' contributions

IS Jeong carried out to make concept and design, or collect data, or analyze and interpret data, and involved in drafting the manuscript or revising it. JS Jeong carried out to make concept and design, or collect data, and involved in drafting the manuscript or revising it. EO Choi participated in its design and involved in drafting the manuscript or revising it. All authors read and approved the final manuscript.

## Pre-publication history

The pre-publication history for this paper can be accessed here:


